# Conception of the Mercury Deposition Coefficient Based on Long-term Stream Intensity Measurements of Mercury Species TGM and TPM

**DOI:** 10.1007/s11270-015-2666-1

**Published:** 2015-11-09

**Authors:** Bartosz Nowak, Marianna Czaplicka

**Affiliations:** Institute for Ecology of Industrial Areas, 6 Kossutha Str., 40-844 Katowice, Poland; Institute of Non-Ferrous Metals, 5 Sowińskiego Str., 44-100 Gliwice, Poland

**Keywords:** Mercury deposition coefficient, Atmospheric mercury species, Mercury wet and dry deposition

## Abstract

For many years, atmospheric mercury has been perceived as a global pollutant. Transport of mercury compounds in the atmosphere and its deposition on the earth’s surface is an important issue that requires knowledge regarding the circulation of the various forms of this metal between environmental components. There are many numerical models that can be used to study and image this phenomenon. These models are based on data concerning mercury emission sources, concentrations of this contaminant on modelling areas and meteorological data to assess air mass inflow on a regional and global scale. A method to assess mercury deposition fluxes on a local scale based only on stream intensity analysis of mercury is proposed in this study. Mercury deposition fluxes (bulk) that were assessed by the MDC method at the Zloty Potok station (regional background station for the Silesian Agglomeration) varied from 22.8 μg · m^−2^ · year^−1^ (an 8-month period in 2013) to 54.2 μg · m^−2^ · year^−1^ in 2012. Developing procedures to estimate the mercury deposition coefficient (MDC) is useful in areas where only meteorological parameters and mercury concentrations in the atmospheric air are measured. The obtained deposition coefficient values enable quantification of a selected pollutant concentration and its potential impact resulting from deposition.

## Introduction

There are several reasons that justify environmental mercury as a global concern. The first reason is that once mercury is introduced into the environment, it remains forever and does not degrade; the second reason is that mercury can be transported over a long distance in a simple way due to the physical properties of the metal. Even small amounts of mercury in the environment may cause negative health effects. Therefore, taking action to reduce mercury emission and focusing on expanding the knowledge about mercury circulation in the environment are very important. In the last few years, mercury release, spread and changes in the environment have awakened significant interest, but despite numerous studies regarding these issues, many of the phenomena remain unexplained. Concentrations of total gaseous mercury (TGM) in ambient air in uncontaminated areas of Asia and North America range from 0.52 to 21.03 ng · m^−3^ (Fu et al. 2008; Choi et al. 2009; Nakagawa and Hirooto 1997; Lynam and Keeler 2006; Fu et al. 2009; Mazur et al. 2009; Liu et al. 2002). Additionally, in unpolluted areas in Europe, the TGM contents in ambient air range from 0.66 to 6.20 ng · m^−3^. Average concentrations of TGM in ambient air in uncontaminated areas of Europe range from 1.96 to 33.8 ng · m^−3^ (Kock et al. 2005; Berg et al. 2001; Zielonka et al. 2005; Pyta et al. 2009). Almost all of these previous research articles showed that concentrations of TGM in winter seasons are significantly higher than in summer seasons, with only a few exceptions in coastal zones, such as Cabo de Creus (Spain); Mèze, Thau Lagoon (France); Piran Marine (Slovenia); Neve Yam, Israel; Halifax (Canada) (Marks and Bełdowska 2001; Bełdowska et al. 2006; Ebinghaus et al. 2006) and the Silesian Region of Poland (Nowak et al. 2014). European legislation and internal regulations in various countries outside of the EU devote much attention to this pollutant. Many actions have been specifically directed at reducing mercury emissions into the environment and phasing-out certain mercury-containing products (European Commission 2004; European Parliament 2005). According to EU legislations, mercury should be constantly monitored and is included in directives and protocols, such as the CAFÉ Directive and Protocol on Heavy Metals (Directive 2004/107/EC). In 2014, the Minamata Convention on Mercury was entered, which has been signed by 128 and ratified by 10 countries so far. The main goals of the convention are to provide comprehensive protection of the environment and human health against the release of mercury into the atmosphere, water and soil. The provisions of the agreement govern issues related to extraction of the metal, trade in products containing mercury and use of this metal in products and industrial processes. The Minamata Convention also established principles for safe management of waste containing mercury and also regulated issues related to mercury-contaminated sites (Minamata Convention 2014).

Transport of mercury compounds in the atmosphere and its deposition on the earth’s surface is an important issue that requires knowledge regarding the circulation of the various forms of this metal between environmental components. There are many numerical models that can be used to study and image this phenomenon. These models are based on data about mercury emission sources, concentrations of this contaminant on modelling areas and meteorological data to assess air mass inflow. One of the basic models used to simulate pollutant transport in ambient air is the Advanced Statistical Trajectory Air Pollution model (ASTRAP) (Shannon and Volder 1995). More complex models describing the transport of various forms of mercury that are based on air mass analysis also exist. One of these models is the Regional Lagrangian Model of Air Pollution (RELAMP) (Eder et al. 1986). This model assumes that reactions in the gas phase are very slow, including oxidation and reduction reactions, and therefore, reactions that may occur in raindrops contained within clouds are included in the calculations. All of the numerical models require meteorological and precision data regarding the types of emission sources present in the modelling area (Travnikov 2005; Tsiros and Ambrose 1999; Bullock 2000). These models can describe not only the transport of mercury in the atmosphere but also the wet and dry depositions and amounts of these contaminants exchanged between various elements in the environment. To further understand the principal mechanisms governing mercury dispersion and cycling in the environment, a global observation system for mercury (GMOS) was created. This system was based on the ECHMERIT and GLEMOS global models and Regional Chemistry Transport Models (CTMs). The GMOS system utilizes data from ground-based stations at high altitudes and sea level locations, ad-hoc oceanographic cruises over the Pacific, Atlantic and Mediterranean and free tropospheric mercury measurements. Application of the GMOS can supplement direct measurements of mercury concentrations and deposition levels, providing more comprehensive and detailed information on the global cycle of mercury (Bencardino et al. 2014; Pirrone et al. 2013; Gencarelli et al. 2014). A large number of emission sources, such as point, linear and area sources, as well as natural processes occurring in the environment are so complex that without a detailed inventory of emission sources, the models cannot accurately capture the specificity of the phenomena that can occur on a local scale. Determining the actual mercury inflow at local measurement points is possible based only on analysing the inflow of mercury streams in the immediate area of the tested point. Although there are methods to analyse contaminant stream inflows that include local and regional scales, simple tools for assessing mercury deposition fluxes on a local scale based on commonly available data are still lacking. Developing a method to assess contaminant deposition fluxes on a local scale based only on stream intensity analysis of those pollutants is one of the challenges of environmental engineering. Accordingly, the primary goal of this study was to develop a procedure to determine Hg deposition fluxes on a local scale based on mercury stream intensities measured in local ambient air monitoring programs.

## Experimental

### Total Gaseous Mercury (TGM) Measurements

TGM concentrations in ambient air were measured using a RA-915+ LUMEX analyser (Lumex Analytics GmbH, Naher str., 558 Wakendorf II, Germany). The analyser operation is based on the differential Zeeman atomic absorption spectrometry technique, which is implemented using the direct Zeeman effect (Zeeman atomic absorption spectrometry using high frequency modulation of light polarization, ZAAS-HFM). The analyser was operated in a continuous mode (time of individual measurement 60 s). Air samples were collected at a level of 2.2 m above the ground. A new calibration method based on preparing reference gas samples of mercury vapours in the concentration range of LOQ-67.6 ng · m^−3^ was applied to validate the analytical procedure for detecting mercury vapours in the concentration range that occurs in ambient air (Nowak et al. 2014). The developed analytical procedure can be characterised by the following parameters: detection limit of 0.24 ng · m^−3^, limit of quantification of 0.48 ng · m^−3^, working range from 0.48 to 67.6 ng · m^−3^, linearity of 0.999, repeatability of 5.3 %, recovery from 98.9 to 107.5 % and expanded uncertainty of 19.7 %. Application of this methodology over a long period of time required stable operation of the analyser. At the inlet of the analyser, a fibre filter was used to absorb particles from the air. For the blank signal control, an effective carbon filter (CF 32 A2B2E2K2Hg-P3) was used to adsorb approximately 99.99 % of the mercury vapour present in the air. Using the carbon filter for blank signal control significantly decreased the level of noise and directly affected the accuracy and precision of the analytical procedure. The limits of detection and quantification were also improved by the significantly decreased background noise.

### Total Particulate Mercury (TPM) Measurement

Particulate matter samples were collected on 47-mm Teflon filters (0.45-mm pore size) housed in acid-cleaned Teflon filter packs at a nominal flow rate of 10 l min^−1^ (Zielonka et al. 2005). Next, the ends of the sampling filters were placed into acid-cleaned Petri dishes and stored in a refrigerator. Upon completion of the measurements, the filters were brought to the laboratory for analysis. The Filters and the particulate matter collected on their surfaces were placed into Teflon vessels for mineralization in a microwave oven (Multiwave 3000-Anton Paar, Austria) using concentrated nitric acid and hydrochloric acid (1:1) (Hg ≤ 0.000001 %, pro analysis, Merck, Germany). The concentrations of mercury were determined by the cold vapor atomic absorbtion spectrometry (CV-AAS) method using an RA-915+ analyser equipped with an RP-91 attachment provided by Lumex Ltd. The operation of the analytical system was checked using appropriately prepared calibration solutions with reference material in the concentration range from 0 to 500 ng · l^−1^. The linear correlation coefficient of the calibration curve was *R*^2^ = 0.974. The method detection limit for TPM was approximately 5 pg · m^−3^ for a 24 h sample at the applied flow rates.

### Wet and dry deposition measurements

Rainfall samples were collected on the bulk sampler. The sampler was made with an acid-washed open borosilicate glass bottle and a 30-cm funnel composed of an inert material. The funnel was supported in a thermostatic housing system and the system protected the samples from solar radiation and high temperatures. On the days without rainfall, the dry deposit collected on the open collector was washed with deionized water on the site. After sampling, the filters with dry deposits were placed into acid-cleaned Petri dishes and stored in the refrigerator. The filters with dry deposits were analysed following the same procedure as the TPM filters (Zielonka et al. 2005; Nowak et al. 2013). The wet deposition samples were preserved with 1 ml of stabilizing solution (nitric acid and potassium dichromate; 5 g K_2_Cr_2_O_7_ + 500 ml HNO_3_/1000 ml) and stored in a Teflon bottle in the refrigerator. After the measurements were completed, samples were mineralized in a water bath for 2 h at a temperature of 95 °C using the following solutions: 0.2 ml (25 g · l^−1^) of potassium permanganate, 0.2 ml of nitric acid (concentrated) and 0.5 ml (40 g · l^−1^) of potassium peroxidisulphate. To the obtained solutions, 100 g · l^−1^ of hydroxylamine hydrochloride was added dropwise to remove excess oxidizer. The concentration of mercury was determined by the CV-AAS method based on an RA-915+ analyser equipped with an RP-91 attachment. The analyser was calibrated using a mercury standard reference material in the concentration range from 0 to 300 ng · l^−1^. The linear correlation coefficient of the calibration curve was *R*^2^ = 0.97504. The detection and quantification limits for total mercury in wet deposition samples were measured using ten independently prepared blank samples. LOD and LOQ amounted to 2 and 5 ng · l^−1^, respectively. The repeatability of this method was 9.4 %. The recoveries were 100.4 %. The laboratory glass and other glass equipment that were used in all conducted analyses were washed in a laboratory washer (Miele G7883, Ontario). All calibration solutions and other reagents were prepared with high-purity deionized water, approximately 0.5 μS/cm, Milli-Q (Millipore, Bedford, MA, USA). The nitric and hydrochloric acids used in the analysis showed very low mercury contents (approximately Hg ≤ 0.000001 %); therefore, its impact on the final results was neglected. The results were corrected using triply prepared blank samples.

### Meteorological Data

Meteorological parameters were determined at all measurement sites. The meteorological stations in Katowice and Pszczyna were equipped with ultrasonic anemometers (81000 YOUNG) used to measure wind speed along the three axes *x*, *y* and *z*, which allowed determination of two horizontal velocities and one vertical velocity as well as air temperature and humidity. In other measuring points, meteorological conditions were measured according to the monitoring plan of the Silesian Inspectorate for Environmental Protection.

### Sampling Site

**Fig. 1:**
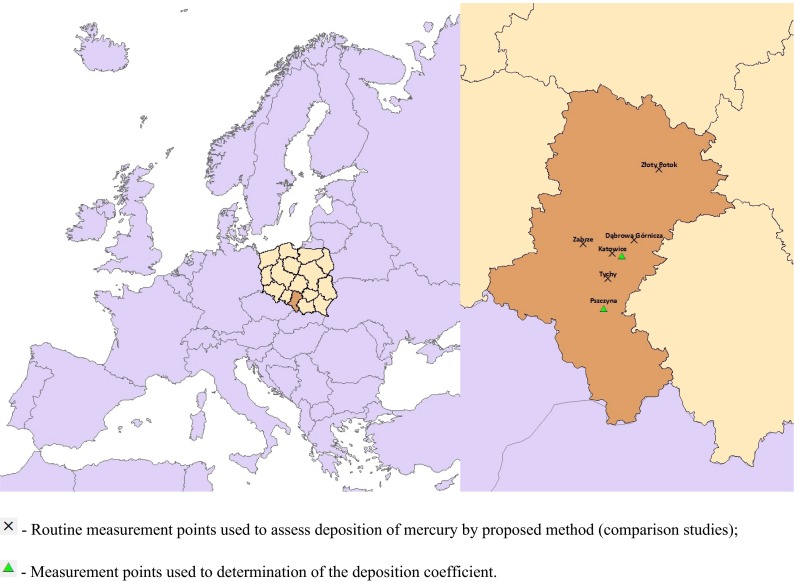
Location of measurement points against the European map and the Silesian Voivodeship map

The measurements were taken in the Upper Silesian region (Southern Poland) from 2008 to 2010. The measuring points were located in the five following Silesian cities (Fig. 1): Katowice, over 300 thousand inhabitants; Dąbrowa Górnicza, approximately 124 thousand inhabitants; Zabrze, approximately 180 thousand inhabitants; Tychy, more than 128 thousand inhabitants; Pszczyna, close to 26 thousand inhabitants. The measurements utilised to determine the deposition coefficient were taken at two measuring points. The TGM and TPM measurements in the atmospheric air and the mercury contents in dry and wet atmospheric precipitations were taken in ten periods (each lasting 21 days) in summer and winter seasons; six measuring periods were performed in Katowice and four in Pszczyna.

### The Mercury Deposition Coefficient Method (MDC)

**Fig. 2:**
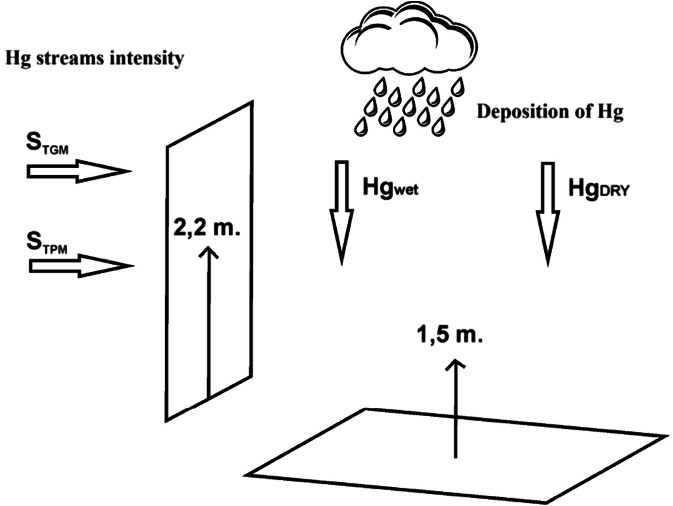
Schematic diagram of mercury deposition coefficient

This work showed that the mercury stream intensity (concentrations in ambient air and meteorological parameters) measured in routine air pollutant monitoring programs can be used to assess mercury deposition. The MDC parameter allows for assessment of mercury deposition. The main goal of this study was to develop a procedure for determination of a Hg deposition coefficient based on analysing mercury stream intensities and compare the obtained results with deposition values measured using chemical analysis. To calculate the deposition coefficient data, the TGM and TPM stream intensities and mercury wet and dry deposition data collected throughout the measuring periods were used (ten measurement campaigns in Katowice and Pszczyna). The coefficient was calculated as a portion of the mercury deposited on the land surface (mercury vertical loads) in the amount of the pollutant transported in the air in the form of TGM and TPM (stream intensity-mercury horizontal loads) within the entire measurement session (see Fig. 2). To determine the TGM and TPM stream intensities, high resolution data regarding the concentrations of TGM and TPM as well as meteorological parameters (wind speed, wind direction) were used.

Stream intensity is defined as a product of the pollutant concentrations and the vector opposite to the wind speed vector. The length of the inflow vector is equal to the intensity of the pollutant stream inflow through the surface that is perpendicular to the wind vector. The inflow vector at the same time indicates the direction of pollutant inflow (TGM and TPM) and their stream intensities.

Based on the TGM and TPM streams intensities, which were measured at a height of 2.2 m during one measurement session, and based on mercury concentration data in wet and dry deposits (deposition values obtained during one measurement session) collected at a height of 1.5 m, mercury deposition coefficients were calculated using the following equation (see below).

$$ \mathrm{M}\mathrm{D}\mathrm{C}={S}_{H{g}_{wet}}+{S}_{H{g}_{dry}}/{\mathrm{S}}_{\mathrm{TGM}}+{\mathrm{S}}_{\mathrm{TPM}};\left[\mathrm{ng}\cdot {\mathrm{m}}^{\hbox{-} 2}\cdot {\mathrm{s}}^{\hbox{-} 1}/\mathrm{ng}\cdot {\mathrm{m}}^{\hbox{-} 2}\cdot {\mathrm{s}}^{\hbox{-} 1}\right] $$

where: ${S}_{H{g}_{Wet}}+{S}_{H{g}_{Dry}}$ is the sum of the wet and dry mercury deposits and *S*_*TGM*_ + *S*_*TPM*_ is the sum of the TGM and TPM stream intensities.

## Results and Discussion

### Overview of Mercury Species Concentrations and Deposits from 2008 to 2010

Upper Silesia is an industrial region located in Southern Poland. In this area, there are 21 mines, which belong to two mining holdings. There are also many mines that do not currently function but contributed to degradation of the natural environment in this region in the past. Many other industries, such as metallurgical, power, engineering and chemical industries are also developed in this area. In the Silesian Voivodeship territory, the atmospheric air pollution situation, especially connected to particles, has been categorised as class C (if the concentration of pollutants exceeds the limit levels as well as the margin of tolerance). Analysing the frequency at which the average annual concentration of PM10 was exceeded showed that at almost all of the measuring stations assessed, the average annual concentrations were much higher than the admissible threshold of 40 μg · m^−3^ (2008/50/EC). The poor air quality is especially related to high levels of low emission in this area that are connected with the burning of solid fuels in domestic heating units. This indicated that during the winter months, the concentrations of mercury in the vapour and adsorbed on the particles are much higher than the concentration in the summer months, which was also reflected in the deposition of this pollutant. The average TGM concentration values obtained during the research conducted at two sites (Katowice and Pszczyna) were very similar to the concentrations previously measured in various locations in Poland and Europe (Kock et al. 2005; Berg et al. 2001; Zielonka et al. 2005; Marks and Bełdowska 2001; Bełdowska et al. 2006; Ebinghaus et al., 2006;Nowak et al. 2014). During the non-heating seasons (summer) that were monitored from 2008–2010, the average TGM concentration in Katowice was 3.49 ± 1.12 ng · m^−3^, but the mean concentration of TGM in Pszczyna was approximately 28 % lower and was approximately 2.53 ± 0.52 ng · m^−3^. In the middle of the heating season (winter), the mean TGM concentration in Katowice was 2.70 ± 0.71 ng · m^−3^. During the heating seasons in Pszczyna, the TGM concentration was approximately 33 % lower and was 1.84 ± 0.41 ng · m^−3^. During the non-heating seasons monitored from 2008–2010, the mean TPM concentration in Katowice was 132.1 ± 107.8 pg · m^−3^ whereas in Pszczyna this value was approximately 97.18 ± 61.33 pg · m^−3^. However, during the heating seasons, the average TPM concentration was 531.7 ± 324.1 pg · m^−3^ whereas in Pszczyna this value was approximately 288.2 ± 165.2 pg · m^−3^. In the winter seasons in Katowice and Pszczyna, the content of TPM in the atmospheric air was several times higher than its content in the summer season. The difference may result from increased combustion of solid fuels in the winter. In the winter season, the consumption of coal significantly increased. This was also confirmed by the approximately 50 % increase in PM10 average daily concentrations in ambient air during the winter season in the Silesian Region. Based on the total annual precipitation amounts in those locations and the average mercury concentrations in wet and dry deposits measured during the conducted studies, the total annual mercury wet and dry depositions were determined. The obtained results are much higher than the literature results (Li et al. 2008; Sakata et al. 2005; Vanarsdale et al. 2005; Gratz et al. 2009). The total average annual values of wet and dry deposition of mercury compounds measured in Katowice from 2008–2010 were 32.1 and 28.2 μg · m^−2^, respectively. The total average annual values of wet and dry deposition measured in Pszczyna were 11.3 and 31.6 μg · m^−2^, respectively. The differences between the observed mercury deposition values may be caused by diverse types of air pollutant emission sources that occur at the measuring points.

### Overview of TGM stream intensities from 2008 to 2010

**Fig. 3:**
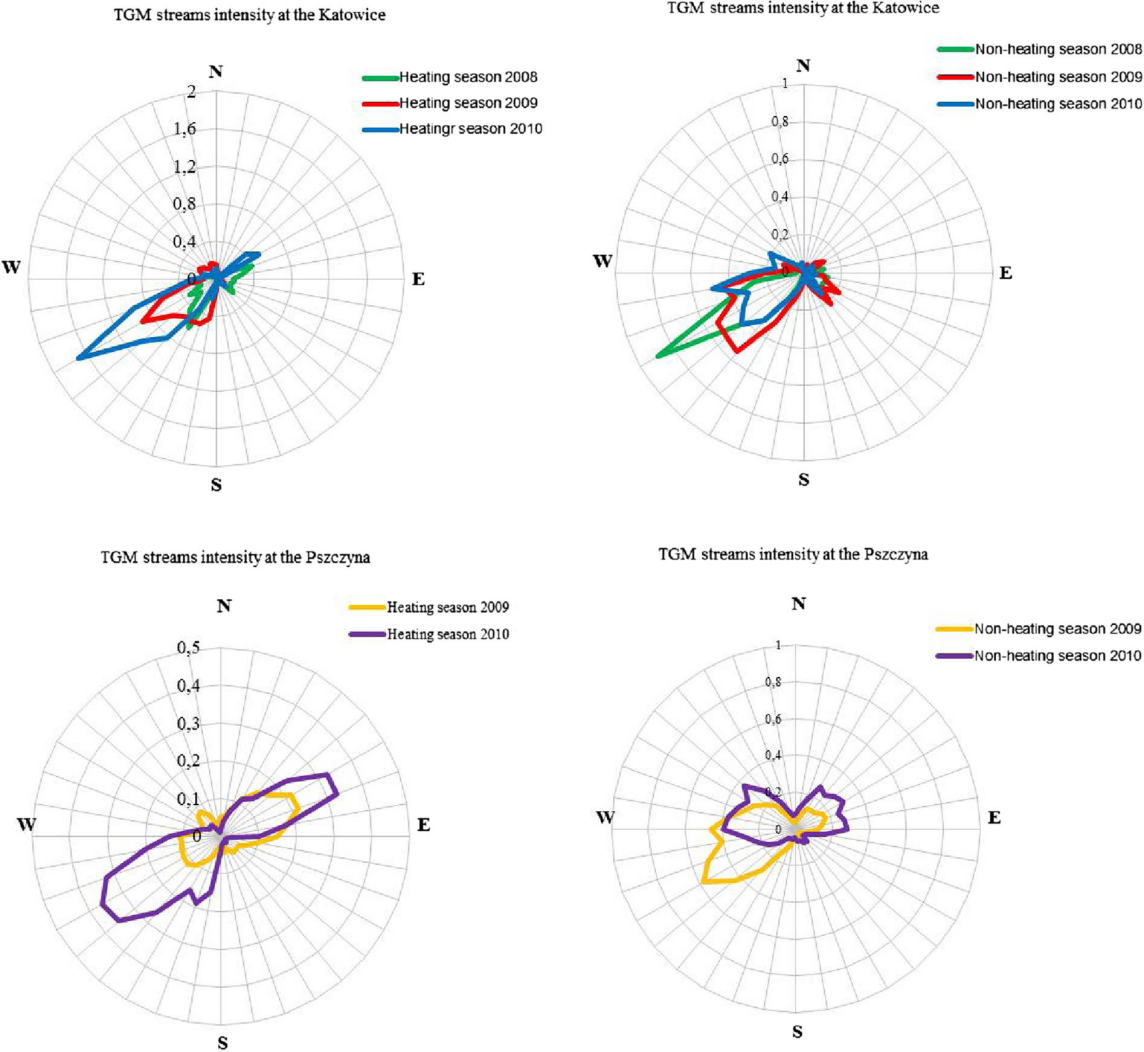
Roses of TGM stream intensity at the monitoring stations in Katowice and Pszczyna during 2008–2010, mg · m^−2^ · 21 days^−1^

To assess mercury deposition using the MDC method, TGM and TPM stream intensity data were needed. Stream intensities of mercury compounds were calculated based on high resolution data regarding the concentration of TGM and TPM as well as meteorological parameters, such as wind speed and wind direction. During the measurement sessions in the non-heating season from 2008–2010, the average TGM stream intensity values were approximately 4.31 mg · m^−2^ · 21 days^−1^, but this value was lower than the result obtained for the heating seasons (6.60 mg · m^−2^ · 21 days^−1^) (see Fig. 3). Additionally, inverse relationships were noted at the air quality monitoring station in Pszczyna. The average TGM stream intensity values during the heating seasons were lower than during the non-heating seasons and amounted to 3.65 and 6.18 mg · m^−2^ · 21 days^−1^, respectively. At both measuring stations in Katowice and Pszczyna as well as in all cases, the average TPM stream intensity values during 2008–2010 were higher during the heating seasons than in the non-heating seasons. At the mercury monitoring station in Katowice, the average TPM stream intensity that flowed through the measuring point from 2008–2010 was 1.17 mg · m^−2^ · 21 days^−1^ in the heating seasons. The result obtained for heating seasons was much higher than the value recorded for non-heating seasons, which was 0.15 mg · m^−2^ · 21 days^−1^. This same trend was observed in Pszczyna from 2009–2010. The TPM stream intensity values for this point in the heating and non-heating seasons were 0.60 and 0.21 mg · m^−2^ · 21 days^−1^, respectively.

### Calculation of the Mercury Deposition Coefficient and Analysis of Parameters Affecting Its Value

In the next stage of this work, mercury deposition coefficients were calculated based on the recorded measurements and proposed methodology (see 2.6). The full data set needed for the MDC calculation is presented in Table 1.

**Table 1:** Set of data necessary to estimate the mercury deposition coefficient and MDC results for the conducted measurement campaigns

Measuring point	Season	Measuring period	TGM streams intensity mg · m^−2^ · 21 days^−1^	TPM streams intensity mg · m^−2^ · 21 days^−1^	Wet deposition μg · m^−2^ · 21 days^−1^	Dry deposition μg · m^−2^ · 21 days^−1^	MDC %
Katowice	Non-heating	20.08–09.09.2008	3.98	0.12	1.43	6.70	0.20
		14.07–04.08.2009	4.82	0.21	2.41	1.23	0.07
		19.05–09.06.2010	4.12	0.12	1.44	0.37	0.04
	Heating	01.12–22.12.2008	4.85	1.40	1.93	1.92	0.06
		09.03–30.03.2009	6.83	1.62	9.17	0.28	0.11
		26.02–18.03.2010	8.11	0.49	0.91	0.55	0.02
Pszczyna	Non-heating	18.05–08.06.2009	6.01	0.22	4.67	2.81	0.12
		02.07–22.07.2010	6.35	0.19	1.20	0.83	0.03
	Heating	20.10–10.11.2009	3.14	0.56	0.76	2.24	0.08
		08.10–29.10.2010	4.16	0.63	0.14	1.53	0.03

The deposition coefficient that was calculated for the measuring stations located in Katowice and Pszczyna in the summer season ranged from 0.03 to 0.12 %, whereas in the winter season these coefficients varied from 0.02 to 0.20 %. At both monitoring stations, the deposition coefficient, which was defined as a portion of the mercury deposited on the land surface (dry and wet) to the amount of the pollutant transported with loads of air in the form of TGM and TPM (stream intensity), did not exceed 0.2 %. As seen, the differences between the obtained MDC values are significant between measurement sessions, and this is important for understanding the causes of these fluctuations in the next section.

### Chemometric analysis

Variation of the TPM and TGM concentrations between the winter and summer seasons and alterations in the meteorological parameters between the seasons contributed to differences in the obtained results. However, additional causes for these fluctuations also exist, which we tried to prove in the next stage of the analysis. These analyses will help determine whether the MDC method can be used to estimate mercury deposition based on commonly available monitoring data regarding mercury concentrations in ambient air.

Meteorological data, such as wind speed, temperature, precipitation height and number of days with precipitation influencing the DMC values were analysed using two independent chemometric techniques i.e. principal component analysis (PCA) and Ward cluster analysis. All of the meteorological data collected during the measurement periods are presented in Table 2. The PCA technique detects existing relations between analysed variables. PCA analysis is based on transforming the originally measured data into a new linear combination of uncorrelated variables, which are called principal components. Clearly interpreting these components (chemical or physical) is very difficult; therefore, the data are appropriately rotated. The purpose of this rotation is to obtain a transparent system of the significance of individual factors characterised by high values of selected variables and low values of others variables. In this analysis, Varimax rotation was used. The analysed data were transformed into a normal distribution. Because each variable was characterised by an individual variation range, it was necessary to standardize the data to correct the proportions, which is called autoscaling (Einax et al. 1997). In the PCA analysis, three factors that explained greater than 65 % of the data variability were analysed.

**Table 2:** Statistical characteristics of meteorological parameters causing MDC fluctuation

Measuring point	Season	Measuring period	Parameter	Wind speed [m · s^−1^]	Temperature [°C]	Precipitation [mm]	Number of days with precipitation	MDC %
Katowice	Non-heating	20.08–09.09.2008	Mean ± SD	0.57 ± 0.27	16.4 ± 2.42	4.00 ± 6.52	5	0.20
			Range	0.2–1.2	11.9–20.9	0.10–15.5		
		14.07–04.08.2009	Mean ± SD	0.60 ± 0.31	19.6 ± 3.64	14.7 ± 17.0	7	0.07
			Min–Max	0.1–1.1	13.2–25.3	0.35–51.8		
		19.05–09.06.2010	Mean ± SD	0.68 ± 0.28	12.9 ± 3.40	6.10 ± 9.57	14	0.04
			Min–Max	0.3–1.3	5.70–20.1	0.16–36.7		
	Heating	01.12–22.12.2008	Mean ± SD	0.91 ± 0.47	1.59 ± 2.33	3.92 ± 4.34	11	0.06
			Min–Max	0.3–2.1	–1.3–6.5	0.30–11.8		
		09.03–30.03.2009	Mean ± SD	1.16 ± 0.65	2.48 ± 3.78	4.84 ± 4.63	14	0.11
			Min–Max	0.3–2.5	–1.9–12.6	1.02–15.3		
		26.02–18.03.2010	Mean ± SD	1.25 ± 0.69	−2.28 ± 3.94	2.88 ± 3.08	11	0.02
			Min–Max	0.1–2.8	−7.8-4.3	0.11–10.4		
Pszczyna	Non-heating	18.05–08.06.2009	Mean ± SD	1.72 ± 0.67	14.3 ± 3.56	3.83 ± 5.12	13	0.12
			Min–Max	0.8–3.1	8.0–21.8	0.25–18.8		
		02.07–22.07.2010	Mean ± SD	1.34 ± 0.48	22.1 ± 3.55	8.21 ± 4.71	4	0.03
			Min–Max	0.7–2.5	15.8–26.1	4.08–13.9		
	Heating	20.10–10.11.2009	Mean ± SD	1.07 ± 0.47	5.05 ± 3.26	2.77 ± 1.94	9	0.08
			Min–Max	0.5–2.2	–1.0–10.7	0.42–6.45		
		08.10–29.10.2010	Mean ± SD	1.47 ± 0.69	5.61 ± 1.75	1.95 ± 2.32	6	0.03
			Min–Max	0.5–3.0	2.3–10.2	0.46–6.54		

**Fig. 4:**
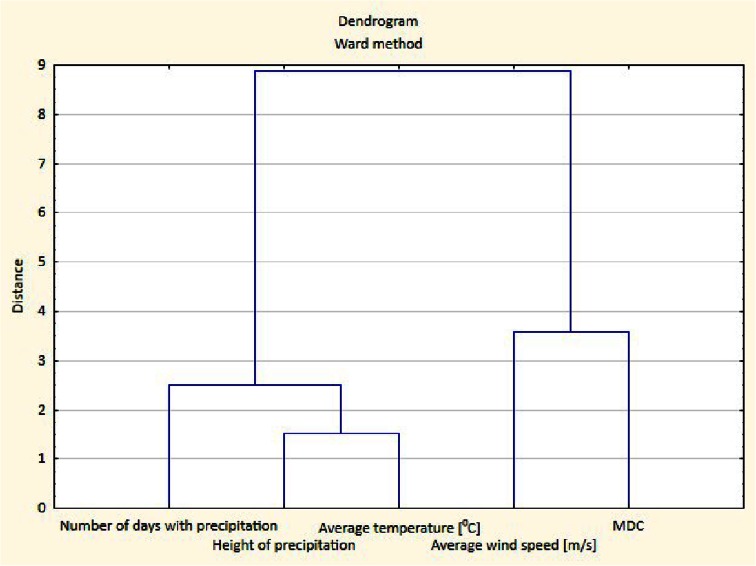
Visualisation of the result of a hierarchical clustering calculation for the meteorological parameters shaping the MDC values

The conducted PCA analysis showed that the first factor explained approximately 35 % of the system variability. Analysing the weight of each factor showed a statistically significant positive correlation between the MDC values and temperature (*r* = 0.80) and precipitation height (*r* = 0.34). This relation was confirmed by the increase of both the MDC and height of precipitation values. This analysis also confirmed that a statistically significant negative correlation existed between the MDC values and wind speed (*r* = −0.70). The PCA method proved that increasing wind speed inhibited deposition (low MDC values). Analysis of the 2nd and 3rd factors did not confirm the presence of other significant dependencies. The results obtained by the PCA method were confirmed using cluster analysis. Cluster analysis revealed the same relationships between the variables that were detected using PCA analysis. The first line of the dendrogram presented below (Fig. 4) showed that a relationship existed between MDC and wind speed, whilst in the second line a relationship between MDC and precipitation height was observed. Chemometric analysis confirmed that a correlation existed between MDC and the meteorological parameters. Thus, using a mercury deposition coefficient method would require similar procedures to be performed as for chemometric analysis. Based on the meteorological data measured during the research studies, differentiation between MDC and the individual ranges of the meteorological parameters, such as wind speed, temperature and precipitation height was completed. The mercury deposition coefficient matrix that resulted from the MDC differentiation assay is shown in Table 3. In this table, the MDC values for the bulk and wet deposition are listed, which depended on the meteorological parameters for which statistically significant correlations were obtained by chemometric analysis. The ranges of meteorological parameters were determined based on the actual data measured during the sessions conducted in Katowice and Pszczyna.

**Table 3:** MDC values by bulk and wet deposition depending on meteorological parameters

MDC _Bulk_ [ng · m^−2^ · s^−1^/ ng · m^−2^ · s^−1^]	MDC _Wet_ [ng · m^−2^ · s^−1^/ ng · m^−2^ · s^−1^]	Percentile	Height of precipitation	Wind speed	Temperature
			[mm]	[m · s^−1^]	[°C]
0.00035	0.00018	25	<1.5	>1.3	<3.1
0.00062	0.00031	50	1.5 ≤ MDC ≤ 3.2	1.1 ≤ MDC ≤ 1.3	3.1 ≤ MDC ≤ 9.3
0.00081	0.00048	75	3.2 < MDC ≤ 3.8	0.7 ≤ MDC < 1.1	9.3 < MDC ≤ 15.9
0.00113	0.00082	90	>3.8	<0.7	>15.9

### Comparison of Mercury Deposition Fluxes Obtained by the MDC and Chemical Analysis Methods

In the last stage of this work, mercury deposition was estimated using publicly available data from monitoring ambient air quality. Deposition estimation was conducted according to the procedure proposed in this paper (MDC method). The obtained results calculated by the MDC method were compared with the data obtained from routine measurements of mercury deposition (chemical analysis). Routine measurement studies (TGM concentration and Hg concentration in wet deposits) to assess mercury deposition using the proposed method and compare the obtained results with real measured values were conducted by the inspectorate for environmental protection from 2008 to 2010 in Dąbrowa Górnicza, Katowice, Tychy and Zabrze (Czaplicka et al. 2009). Based on the TGM concentration data in ambient air and wind field data, mercury vapour stream intensities at four measurement stations were calculated. In the next step, statistical characteristics of meteorological parameters were appropriately selected for MDC analysis, as shown in Table 4. When all relevant parameters e.g. TGM stream intensity, were collected, mercury wet deposition was calculated (individual meteorological parameters at each measuring point were chosen) (see Table 4). Mercury deposition fluxes that were assessed by the MDC method in 2012 at the four measuring stations ranged from 29.2 (Dąbrowa Górnicza) to 42.5 μg · m^−2^ · year^−1^ (Tychy). Similarly, values measured by chemical analysis in these places varied from 29.0 (Dąbrowa Górnicza) to 44.5 μg · m^−2^ · year^−1^ (Tychy) (Czaplicka et al. 2009). The percent difference between the results obtained by the two methods fluctuated from −4.3 to 11 %. The highest mercury deposition flux deviation was approximately 4 μg · m^−2^ · year^−1^, which was noted at the Katowice station, whilst the smallest difference of approximately 0.2 μg · m^−2^ · year^−1^ was observed at the Dąbrowa Górnicza station. The absolute percent difference between the two deposition estimation methods for the four points was 5.3 %.

**Table 4:** Comparison of deposition values estimated by MDC method and measured deposition values for the four monitoring stations in 2012

Measuring points	Hight of precipitation [mm]	Wind speed	Temp	MDC	Assessment values of deposition by MDC method [μg · m^−2^ · year^−1^]	Measured values of deposition by chemical analysis [μg · m^−2^ · year^−1^]	Percentage difference [%]
		[m · s^−1^]	[°C]	(Wet)			
				[ng · m^−2^ · s^−1^/ ng · m^−2^ · s^−1^]			
Zabrze	1.7	1.26	6.9	0.00031	37.8	36.0	5.1
Katowice	1.7	0.95	7.9	0.00048	39.7	35.7	11.0
Tychy	1.7	1.0	7.6	0.00048	42.5	44.5	−4.3
Dąbrowa Górnicza	1.7	1.1	6.1	0.00031	29.2	29.0	0.8

### Assessment of Mercury Deposition Fluxes by the MDC Method

The mercury deposition assessment also used TGM concentrations and meteorological data obtained from 2010–2013 that were measured under the national monitoring program by the Silesian Inspectorate for Environmental Protection at the Zloty Potok station. This station is located in a rural area and provides regional background for the Silesian Agglomeration. To estimate the deposition of mercury in Zloty Potok, appropriate MDC values were chosen according to Table 3, and the meteorological data presented below were also utilised (see Table 5). Estimated mercury deposition fluxes (bulk) at the Zloty Potok station varied from 22.8 μg · m^−2^ · year^−1^(an 8-month period in 2013) to 54.2 μg · m^−2^ · year^−1^ in 2012. The obtained results can be compared with mercury deposition values measured between April 2008 and September 2009 at eight monitoring locations in the Silesian Agglomeration territory (Zabrze, Bytom, Radzionków, Katowice, Dąbrowa Górnicza Tychy) (Czaplicka et al. 2009). The mercury deposition results were significantly different for individual months depending on the precipitation height. For the Silesian Agglomeration, the mercury wet deposition during an 18 month measuring period ranged from 32 to 48 μg · m^−2^. Recalculating these values for a full year generated mercury deposition fluxes between 21.3 and 32.0 μg · m^−2^. Correlating the measured values with the values calculated by the MDC method at the Zloty Potok monitoring station in 2012 showed that the obtained results are comparable.

**Table 5:** Comparison of estimated deposition values by MDC method and measured deposition values for the Zloty Potok monitoring stations during 2010–2013

Measuring period	Hight of recipitation [mm]	Wind speed	Temp	MDC	MDC	Assessment values of deposition by MDC method (bulk) [μg · m^−2^ · year^−1^]	Assessment values of deposition by MDC method (wet) [μg · m^−2^ · year^−1^]
		[m · s^−1^]	[°C]	(bulk)	(wet)		
				[ng · m^−2^ · s^−1^/ ng · m^−2^ · s^−1^]			
June–December 2010	2.5	1.69	8.9	0.00062	0.00031	36.4	18.2
January–June 2011	1.4	1.71	5.7	0.00035	0.00018	27.5	14.1
January–December 2012	1.8	1.78	8.2	0.00062	0.00031	54.2	27.1
January–August 2013	1.0	1.90	8.8	0.00035	0.00018	22.8	11.7

## Conclusions

The research presented above demonstrated that in areas where only meteorological parameters and mercury concentrations in atmospheric air are measured, it is possible to increase the amount of information about the processes of environmental pollution with mercury and what can be done using the MDC method. The proposal to calculate the deposition coefficient using TGM and TPM stream intensities is a new solution. This is especially important when we want to assess to what extent the process of mercury deposition contributes to the pollution of soil, vegetation and surface waters. The proposed deposition coefficient allows quantification of a selected pollutant concentration and its potential impact as a result of deposition. Further studies on the deposition coefficient may contribute to developing methods to estimate the impact of ambient air pollutants on other environmental components based on analyses of the pollutant stream intensity. Additionally, we may be able to determine the direction from which the pollutant (risk) was derived. Further development of this method may lead to identification of mercury emission sources.
